# Purification and characterization of aspartic protease from *Aspergillus niger* and its efficient hydrolysis applications in soy protein degradation

**DOI:** 10.1186/s12934-023-02047-9

**Published:** 2023-03-03

**Authors:** Mengyuan Wei, Pengcheng Chen, Pu Zheng, Xiumei Tao, Xiaowei Yu, Dan Wu

**Affiliations:** 1 The Key Laboratory of Industrial Biotechnology, Ministry of Education, School of Biotechnology, Jiangnan University, Wuxi, 214122 China; 2 State Key Laboratory of Food Science and Technology, Jiangnan University, Wuxi, China

**Keywords:** Aspartic protease, *Aspergillus niger*, Heterologous expression, *Pichia pastoris*, Soybean isolate protein, Protein degradation

## Abstract

**Background:**

Adding acid protease to feed can enhance protein digestibility, boost feed utilization, and stimulate the growth of animals in breading industry. In order to obtain an acid protease with high hydrolysis efficiency to plant protein, in this study, an aspartic protease from *Aspergillus niger* was heterologous expressed in *Pichia pastoris (P. pastoris)*. The enzymatic properties and application in soybean protein degradation were also studied.

**Results:**

In our investigation, the high aspartic protease (Apa1) activity level of 1500 U/mL was achieved in 3 L bioreactor. After dialysis and anion exchange chromatography, the total enzyme activity and specific enzyme activity were 9412 U and 4852 U/mg, respectively. The molecular weight of the purified protease was 50 kDa, while the optimal pH and temperature were 3.0 and 50 °C, respectively. It was stable at pH 2.0–5.0 and 30–60 °C. Apa1 was used to hydrolyze soybean isolate protein (SPI) at 40 °C and pH 3.0, and a high hydrolysis degree (DH) of 61.65% was achieved. In addition, the molecular weight distribution of SPI hydrolysis products was studied, the result showed that the hydrolysis products were primarily oligopeptides with molecular weights of 189 Da or below.

**Conclusions:**

In this study, Apa1 was successfully expressed in *P. pastoris* and high expression level was obtained. In addition, the highest protein hydrolysis rate to SPI degradation so far was achieved. The acid protease in this study provides a new protease that is suitable for the feed industry, which will be very helpful to improve the feed utilization and promote the development of the breeding industry.

**Supplementary Information:**

The online version contains supplementary material available at 10.1186/s12934-023-02047-9.

## Introduction

Proteases are a highly complex group of enzymes with different substrate specificities, catalytic mechanisms, and activity sites [1]. They are classified into six mechanistic classes, namely, cysteine proteases, serine proteases, aspartate proteases, metalloproteinases, threonine proteases, and unknown type of proteases. Each class has a set of characteristic functional amino acid residues, which are arranged in specific configurations to form active sites [2]. Aspartate proteases (E.C.3.4.23) are found mainly in animal cells, plants, mycobacteria, and yeast [3]. This enzyme is an endopeptidase with a molecular weight in the range of 30–45 kDa. Aspartic proteases have been usually called acid proteases since they showed optimal activity in the acidic pH range (pH 3.0–5.5). Most of them are stable within the acidic to neutral pH range, making them valuable for applications in many industrial processes such as food processing [4], the breading industry [5], leather manufacturing [6], medical applications [7], etc.

Aspartic proteases are produced mainly by filamentous fungi, specifically *Aspergillus*. Fungi, such as *Aspergillus niger* [8], *Aspergillus oryzae* [9], and *Aspergillus fumigatus* [10], are widely applied for aspartate protease production. In industrial application, the *Aspergillus niger* is the species that mainly used for producing aspartate protease [11]. *Aspergillus niger* have been certified as Generally Recognized as Safe (GRAS), indicating that their products are safe for human and animal consumption.

Aspartate proteases are one of the most innovative products for improving breading industry efficiency. Because of their hydrolytic properties, they can promote the absorption of amino acids, thus saving feed costs significantly [12]. Feed enzymes’ catalytic reactions occur mainly in animals’ guts. The conditions in the animal gut, with a temperature of 40 °C and a pH range of 1.5–6, are appropriate for protein degradation by aspartate protease [13–15]. In some young animals’ feeding processes, conventional feeds, particularly high-protein feeds, frequently lead to incomplete digestion and low absorption of nutrients, which can easily result in digestive disorders in the intestinal tract of animals and thus usually in the animals’ death [16–18]. Soybeans contain 35–50% protein (on a dry weight basis) and are very economical compared to other forage proteins, this advantage cause it widespread used in food and feed worldwide. Protein hydrolysis products was produced in the process that the protein macromolecules in soybeans were decomposed into lower molecular-weight peptides and free amino acids, which is an efficiency way to enhancing the digestibility of animal feeds [19]. The degradation products of aspartate protease are mainly small peptides with low molecular weight and some free amino acids, among which bioactive peptides have the advantages of anti-cancer activity and low cholesterol properties [20, 21]. Therefore, the addition of a certain amount of aspartic protease in the feed is conducive to the complete degradation of protein in the gastrointestinal tract of animals, promoting the digestion and absorption of low-molecular-weight peptides, free amino acids, and other nutrients and also significantly improving feed utilization and reducing the gastrointestinal stimulation for feeding in young animals.

*P. pastoris* is widely used as an heterologous expression host because of its endotoxin-free use, high exogenous-protein expression, easy product purification, low production cost, and suitability for high density fermentation [22, 23]. There are many examples of aspartic proteases successfully expressed in this host, including Bsapa from *Bisporomyces* MEY-1 [24], RmproA from *Talaromyces leycettanus* [25], and MCAP from *Mucor circinelloides* DSM 2183 [26]. But there are few research on protease hydrolysis of soy protein, and it is an inevitable problem that the DH of soy protein is relative low (generally 5–8%) [27–29]. These poor utilization rates of nutrients limit large-scale application of proteases in the breeding industry.

In this study, the main objectives were to determine the biochemical characteristics of aspartate protease Apa1 from *A. niger* expressed by *P. pastoris* for the first time, including the optimum pH and temperature for activity and activity stability, as well as the kinetic parameters *K*_m_ and *V*_max_. After biochemical characterization, the application of the protease to SPI hydrolysis under conditions that were close to the gastrointestinal environment of animals was evaluated.

## Materials and methods

### Strains, culture media, and reagents

In this study, *Escherichia coli* (*E. coli*) JM109 was used in cloning procedure, and *P. pastoris* X33 was used as the host strain. Both *E. coli* and *P. pastoris* X33 were reserved in our laboratory. Prime Star Max DNA Polymerase, Sac I, Sal I endonuclease and other restriction endonucleases were bought from Takara Biotech (Dalian) Co., Ltd. rTaq DNA polymerase, One-step cloning kit were bought from Nanjing Vazyme Biotech Co., Ltd. Plasmid extraction kit and PCR product purification kit were bought from Shanghai Generay Biotech Co., Ltd. Protein electrophoresis SDS-PAGE kit was bought from Beyotime Biotechnology Co., Ltd. Bleomycin (zeocin) was bought from Beijing Solarbio Technology Co., Ltd. Yeast extract and tryptone were bought from Oxoid Ltd. Gene synthesis and PCR primers were made by Scientific Talen-bio Scientific (Shanghai) Co., Ltd..

*E. coli* JM109 was incubated in Luria-Bertani medium (yeast powder 5 g/L, tryptone 10 g/L, NaCl 10 g/L) with shaking at 37 °C and 220 r/min. All *P. pastoris* protocols and media were performed and manufactured in accordance with the Invitrogen (California, United States) manual. All the other chemical reagents used were analytical grade.

### Codon optimization and construction of recombinant plasmids

The aspartate protease gene was obtained from NCBI (National Center for Biotechnology Information) with the sequence number XP_001401093.1 and base-substituted using SnapGene 3.2.1 according to *P. pastoris* codon preference. The optimized gene (*apa1*) was synthesized by Sangon (Shanghai, China). The synthesized *apa1* gene was amplified using PCR with forward primer (GCTCCAGCTCCAACTAGAAAG) and reverse primer (AGCTTGAGCAGCAAAACCC) specific to its sequence. The truncated *apa1* gene without the signal peptide coding sequence was cloned into the pPICZαA vector using a one-step cloning ligation. The ligated product was identified by agarose gel electrophoresis and purified by a gel recovery kit. The pPICZαA/*apa1* plasmid was validated by sequencing.

### Transformation and screening of E. coli and P. pastoris

Recombinant expression plasmids were transformed into *E. coli* JM109 competent cells by heat excitation and cultured overnight in Luria-Bertani (LB) plates containing 1% zeocin antibiotics. Positive colonies were screened by colony PCR, and the plasmids were verified by sequencing.

The correctly expression plasmids were linearized with Sal I endonuclease, and the DNA was purified using a PCR product recovery kit and transformed into *P. pastoris* X33 competent cells [30, 31]. After quickly adding 1.0 mL of pre-chilled 1.0 M sorbitol, the cells were incubated at 30 °C, 220 r/min for 2 h before being inoculated in yeast extract peptone dextrose (YPD) plates containing 0.1% zeocin antibiotic, and randomly selecting single colonies for shake flask fermentation re-screening. The bacteria were incubated in YPD liquid medium at 30 °C and 220 r/min for 24 h, and the organisms were centrifuged and transferred to a yeast extract peptone (YP) medium for induction cultivation by adding 1% methanol every 12 h. After 72 h, the fermentation was stopped, and the supernatant collected by centrifugation was referred to as crude enzyme.

### High density fermentation of recombinant strains

The screening strains were subjected to high density fermentation according to Jia et al. [32]. The recombinant strain was cultivated in a 300 mL flask containing 100 mL YPD medium, incubated at 30 °C and 220 r/min in a shaker until OD_600_ = 12–15, and then the culture was inoculated into a 3 L fermenter containing 900 mL BSM medium. The fermenter was maintained at 30 °C, pH 5.0 (controlled by ammonia), 2–5 vvm of aeration, and 400–800 r/min of stirring speed. When the strain completely consumed the glycerol in BSM (the DO value increased abruptly), 500 g/L glycerol (containing 12% PTM1) was fed until OD_600_ reached 100. After 2 h starvation, the induction initiated by feeding methanol controlled by DO and fermenter temperature was set at 28 °C, pH 5.0–5.5, stirring speed at 800 r/min, DO value at 20–25%. The fermentation samples were taken every 12 h until the enzyme activity no longer increased significantly. Finally, the fermentation supernatant was collected by centrifuging at 4 °C for 10 min at 8000 r/min and then kept at 4 °C.

### Protease activity assay and protein concentration determination

Aspartate protease activity was determined using the forint-phenol method [33] and 1% casein solution as the substrate. In a water bath of 40 °C, 1 mL of preheated casein substrate was mixed with 1 mL of the appropriate concentration of enzyme solution (lactic acid buffer pH 3.0 dilution) for 10 min. The reaction was stopped by adding 2 mL of a 0.4 mol/L solution of trichloroacetic acid for 10 min. After centrifugation at 10000 r/min for 5 min, 500 μL supernatant was added into 2.5 mL sodium carbonate solution (0.4 mol/L) and 500 μL Folin reagent. Then the mixture was put into a water bath at 40 °C for 20 min after shaking well and was cooled immediately to room temperature. The absorbance value at 680 nm was determined. One unit of enzyme activity was defined as the amount of enzyme required to hydrolyze casein and release equivalent of 1 μg of phenolic amino acids within 1 min of reaction.

For protein concentration determination, the bovine serum protein was the standard protein [34], and the protein concentration was determined according to the Lowery method [35].

### Purification of protease

Apa1 crude enzyme solution was purified using the anion exchange principle on an AKTA purification system. The crude enzyme solution was fully dialyzed in phosphate buffer (pH 5.8). The anion exchange column HiTrap^™^ 1 mL Q HP was equilibrated with phosphate buffer, then the dialyzed enzyme solution was loaded into the column and eluted with a gradient at a flow rate of 0.3 mL/min. The fractions were detected by UV absorbance at 280 nm for collection. All graded collection fractions were taken and put through SDS-PAGE protein electrophoresis according to articles [36] to determine the size of the purified enzyme bands and their aspartate protease activity.

### Effects of pH and temperature on activity and stability of protease

For determination of Apa1 optimal temperature, the enzyme activity was measured at pH 3.0 in 30–70 °C. For determination of optimal pH, different buffer systems such as lactic acid-sodium lactate buffer pH 2.0–3.0, acetic acid-sodium acetate buffer pH 4.0, and disodium hydrogen phosphate-citrate buffer pH 5.0–8.0 were prepared, and the corresponding enzyme activities were measured at 40 °C. For determination of thermal stability, the enzyme solution was diluted to appropriate concentration in a water bath at various temperatures for 20 min, then cooled rapidly. The enzyme activity was determined according to the above procedure. For determination of pH stability, the enzyme solution was incubated in various pH buffers for 60 min, and then the enzyme activity was measured. The relative enzyme activities were determined by making the highest enzyme activity equal to 100%.

### Kinetic determination of kinetic parameters

The *K*_m_ and *V*_max_ values for the purified aspartate protease were determined with different casein substrate concentrations (0.2–3.0 g/L) at pH 3.0 and 40 °C. A double reciprocal plot was used for estimating *K*_m_ and *V*_max_.

### Hydrolysis of soy protein by aspartate protease

The protein hydrolysis study simulates the animals’ gastrointestinal environment and reflects the hydrolysis of soybean isolate protein in animals’ gut [37]. The enzyme solution was dialyzed overnight in a lactic acid buffer (pH 3.0) and hydrolyzed different concentrations of SPI. Hydrolysis degree was measured by the o-phenylene formaldehyde method (OPA) [34]. Dissolve SPI completely in lactic acid-sodium lactate buffer, pH 3.0, and mix 2.5 mL SPI and 2.5 mL dialysis enzyme solution in a water bath shaker at 40 °C. Samples were taken at intervals until the reaction was completed. The following Eqs. (1)-(3) were used to calculate the DH of samples:

1 $$ DH \, = \frac{h}{h}_{tot} \times \, 100\% $$

2 $$ h \, = \frac{{SerineNH_{2} - \beta }}{\alpha }{{meqv} \mathord{\left/ {\vphantom {{meqv} g}} \right. \kern-0pt} g} $$

3 $$ SerineNH_{2} = \frac{{OD_{sample} - OD_{blank} }}{{OD_{{{\text{standard}}}} - OD_{blank} }} \times 0.9516{{meqv} \mathord{\left/ {\vphantom {{meqv} l}} \right. \kern-0pt} l} \times \frac{{0.1 \times 100{l \mathord{\left/ {\vphantom {l g}} \right. \kern-0pt} g}}}{X \times P} $$

where h is the number of peptide bonds cleaved, and *h*_*tot*_ is the total number of peptide bonds. As reported, *h*_*tot*_ was calculated to be 7.8 meqv/g for SPI. *SerineNH*_*2*_ is meqv serine NH_2_/g protein, *X* is the sample’s mass, and *P* is the sample’s protein concentration. For SPI, *α* and *β* were reported to be 0.970 and 0.342, respectively.

The reaction solutions of samples were subjected to SDS-PAGE protein electrophoresis for the preliminary analysis of SPI hydrolysis process. The reaction solution was taken until the DH no longer increased significantly, and the resulting peptides and their molecular weights (Mw) were determined by HPLC (Waters 2695). The column was TSK gel 2,000 SW_XL_ 300 mm*7.8 mm with the mobile phase acetonitrile/water/trifluoroacetic acid ratio of 40/60/0.1 (v/v). The elution was carried out at 30 °C with a flow rate of 0.5 mL/min and UV 220 nm. The standards (purchased from Sigma) used for the molecular weight calibration curve were cytochrome C (Mw 12,384), peptidase (Mw 6500), bacillus peptide (Mw 1422), ethane-ethane-tyrosine-arginine (Mw 451), and ethane-ethane-ethane (Mw 189).

## Results and discussion

### Synthesis of codon-optimized gene and expression in P. pastoris

Analysis of the nucleotide sequence of *apa1* revealed that the codons of a few amino acids do not correspond to *P. pastoris* codon preference. These codons were substituted with high-frequency codons to increase protein expression efficiency in *P. pastoris*. A total of 111 nucleotides were substituted in the optimized gene, which shared 90% of its nucleotide sequence with the original gene (Additional file 1: Fig S1).

The pPICZαA/*apa1* plasmid was validated by sequencing, linearized, and then transformed into *P. pastoris* X33. Electrophoresis of the amplified target gene bands (1122 bp) revealed that their sizes were correct (Additional file 1: Fig S2), demonstrating that the *apa1* gene was successfully inserted into the transformant’s genome.

### Recombinant expression of Apa1 in a 3 L bioreactor

**Fig. 1:**
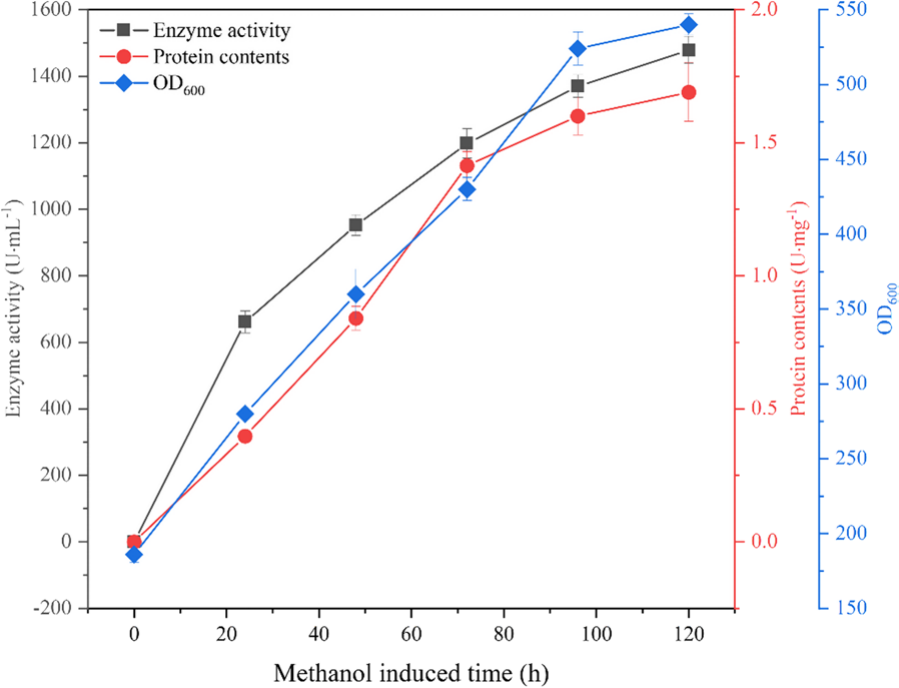
Process parameters for recombinant-bacteria Apa1 fermentation for a 3 L Fermenter

Multiple colonies were randomly selected from YPD plates of transformed *P. pastoris* and then re-screened in shake flasks. The strain with the highest aspartate protease enzyme activity was identified after 72 h of fermentation induced by 1% methanol, with an enzyme activity of 157.93 U/mL. As shown in Fig. 1, the strain was used for scaled-up fermentation in a 3 L laboratory bioreactor. The protein content increased significantly along with fermentation. At 120 h of induction, the protein content, enzyme activity, and OD_600_ values no longer increased significantly, and the highest OD_600_ value reaching 540. The highest enzyme activity reached 1500 U/mL, which is 9.36 times greater than the amount in the shake flask.

### *Puri*fication of recombinant* Apa1*

**Fig. 2:**
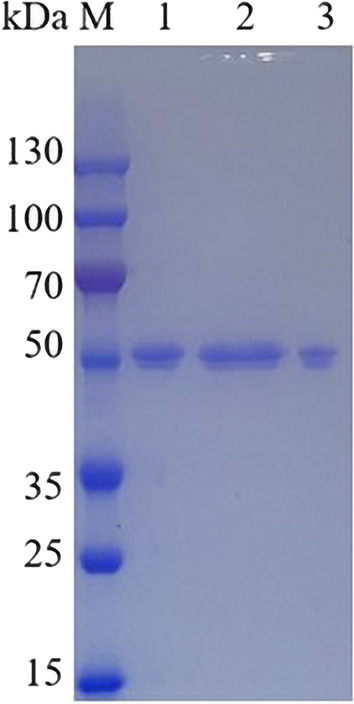
SDS-PAGE analysis of purified Apa1 protein. *M* Molecular-weight standard proteins. 1: purification fraction 5a5. 2: purification fraction 5a6. 3: purification fraction 5a7

The supernatant of the 3 L bioreactor was purified using an anionic column after overnight dialysis. One prominent protein peak was observed, and enzyme activity was detected in purification perforated plate receiver fractions 5a5, 5a6, and 5a7. After purification, the overall enzyme activity was 9412 U, and the specific enzyme activity increased to 4852 U/mg, which was 2.24 times higher than that of the crude enzyme solution after dialysis, and the final yield rate was 72%. As shown in Fig. 2, SDS-PAGE indicated that the purified enzyme solution was a clear single band with an apparent molecular weight of around 50 kDa. The theoretical molecular weight of this protein is 39.3 kDa, which is lower than the apparent molecular weight, and it was speculated that the glycosylation reaction may have occurred [2, 24, 38]. Glycosylation frequently occurs in yeast expression systems, and previous studies have shown that glycosylases exhibit better thermal stability [39–41]. All purification process parameters were shown in Table 1.

**Table 1:** Apa1 protease purification results summary

Apa1	Total volume (ml)	Total protein (mg)	Total enzyme activity (U)	Specific activity (U/mg)	Purification multiple	Recovery rate %
Crude enzyme solution after dialysis	5.00	5.99	12989	2169	1.00	100
Pure enzyme	2.00	1.94	9412	4852	2.24	72

### Enzymatic properties of Apa1

**Fig. 3:**
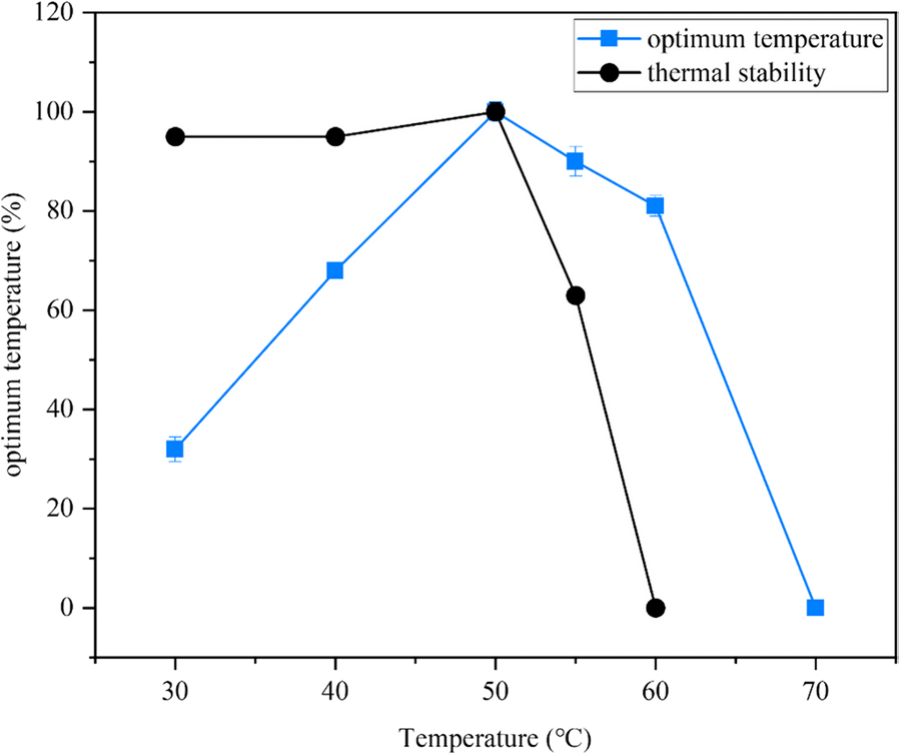
Optimal temperature and thermal stability of aspartate protease Apa1. Relative activity is expressed as a percentage relative to the highest activity (highest activity is 100%)

The optimum temperature for Apa1 in lactic acid buffer (pH 3.0) was 50 °C. The enzyme retained 81% activity at 60 °C, but we could not detect activity at 65 °C (Fig. 3). When Apa1 was incubated at 30–50 °C for 3 h, the enzyme exhibited high stability, while the enzyme activity declined significantly beyond 50 °C, and it was completely inactivated after 10 min of incubation at 60 °C (Fig. 3). The effect of temperature on Apa1 is consistent with the general properties of majority of reported aspartic proteases, such as the aspartic protease PsAPA from *Penicillium spp* XT7, which has an optimum temperature of 30 °C and complete inactivation at 50 °C [38]. Aspartic protease from *Aspergillus foetidus* has an optimal temperature of 55 °C, and the enzyme activity is lost by 50% at 65 °C [42]; the optimal temperature of aspartic protease from *Aspergillus niger* BCRC 32720 is 50 °C, whereas the activity rapidly drops at 65 °C [43].

**Fig. 4:**
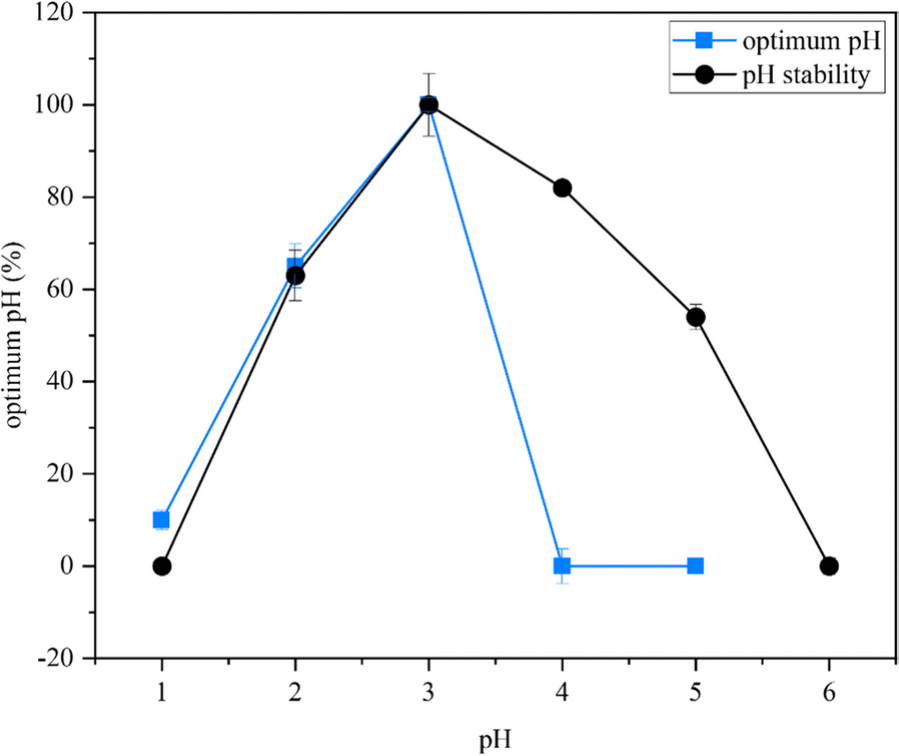
Optimal pH and pH stability of aspartate protease Apa1. Relative activity is expressed as a percentage relative to the highest activity (highest activity is 100%)

Apa1 exhibits maximum activity at pH 3.0 and maintained more than 50% activity over 60 min incubation at pH 2.0–5.0 (Fig. 4), showing that Apa1 had good enzyme activity under acidic conditions. The recombinant Apa1 could be widely used in the future in many industries such as food processing and breeding industries, where its acidic application environment will help reduce energy usage and inhibit microbial deterioration [44].

### Kinetic parameters

The Lineweaver-Burk model was used to demonstrate the enzyme kinetic data by using 0.2–3.0 g/L casein as a substrate. The *K*_m_ and *V*_max_ values determined by the software Origin 2021 were 2.44 mg/mL and 83.54 U/mg, respectively. This result is similar toother recombinant aspartic proteases, such as the *K*_m_ against casein of aspartic proteases PepAb (2.5 mg/mL), aspartic proteases PepAc (1.9 mg/mL) [28], aspartic proteases MpiAP1 (2.0 mg/mL), and the aspartic proteases MpiAP2 (3.5 mg/mL) [45]. These results indicate that the recombinant protein was successfully expressed in *P. pastoris*.

### Hydrolysis of SPI with Apa1

**Fig. 5:**
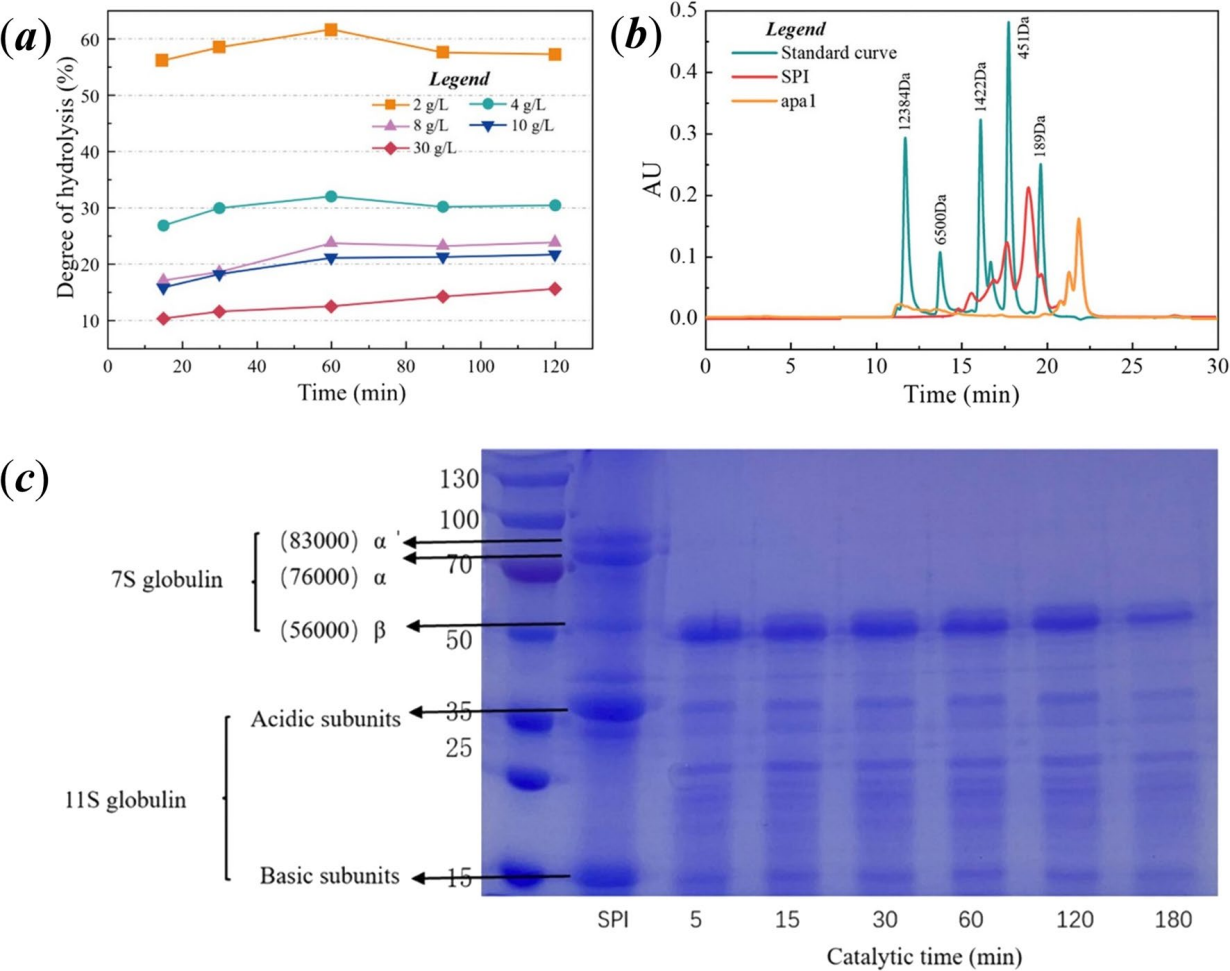
Analysis of the DH and hydrolysis products of Apa1-catalyzed soybean isolate protein. **a**: Hydrolysis curves with time for different concentrations of soybean isolate as substrate. **b**: HPLC chromatography of hydrolysis products when hydrolyzed to a constant DH. **c**: SDS-PAGE analysis of hydrolysis products upon hydrolysis to a constant DH

Apa1 was applied to protein hydrolysis reaction with various concentrations of SPI. The hydrolysis reaction generally reached its maximum when it was carried out for up to 60 min, as shown in Fig. 5a. By measuring the DH of the reaction, we determined that the concentration of 2 g/L SPI was degraded with a maximum DH of 61.65%, while the concentration of 4 g/L SPI also reached 32% DH. A commercial enzyme was randomly selected and compared with the hydrolysis reaction under the same conditions described above. Apa1 was 11.89 times more effective than the commercial enzyme and better than majority of aspartate proteases reported, such as the DH of SPI by pepsin, which can reach up to 7.52% [27], the DH of aspartic proteases PepAb, PepAc, and PepA from *Aspergillus niger* F0215, which are 7.6%, 6.9%, and 8.7%, respectively [28], and the DH of aspartic proteases FAP can about reach up to 6.2% [29]. Apa1 was the best acid protease for SPI degradation reported so far.

SPI is mainly composed of two subunits: 7S globulin (β-conarachin) and 11S globulin (soybean globulin); 7S globulin is primarily made up of α′, α, and β subunits, and 11S globulin is primarily made up of acidic and basic subunits [33]. As shown in Fig. 5c, The SDS-PAGE results demonstrated that the α' and α subunit portions of 7S globulin in SPI (83 and 76 kDa) were hydrolyzed by Apa1. In contrast, the β subunit band of 7S globulin thickened, indicating that Apa1 did not hydrolyze the β subunit of 7S globulin. The hydrolysis of Apa1 significantly decreased the acidic and basic subunits contents of 11S globulin in SPI, and the hydrolysis reaction was accompanied with the production of an approximately 25 kDa hydrolysis product band. A degradation study of SPI using trypsin by Yoshiro Kamata et al. revealed that hydrolysis of 7S globulin generated a stable fragment (25–30 kDa) [46]. In the study by K. Tsumura et al. the 11S globulin fraction of natural soy protein isolate was selectively hydrolyzed by pepsin in the pH range of 1.5–2.5, while the 7S globulin fraction was not easily digested by pepsin [47]. In the study by Chun Cui et al., they used pepsin, which completes the degradation of 11S globulin and has little hydrolysis effect on 7S globulin [27]. The above results indicate that aspartate protease has cleavage specificity, and Apa1 in this study also exhibited a significant degradation effect and hydrolysis specificity for SPI.

When the hydrolysis reached a state of equilibrium, the peptide spectrum analysis revealed that the hydrolysis products of Apa1 were composed of oligopeptides with molecular weights below 189 Da, as shown in Fig. 5b (the graph is a small-molecule peptide chromatogram, hence, the undegraded subunit cannot be detected). In the study by Peng Song et al., PepAb and PepAc in Saccharomyces cerevisiae hydrolyzed SPI released oligopeptide polymers (Mws) of 189–1450 Da (about 2–11 amino acid residues), while PepA tended to release oligopeptides with a higher molecular weight [28]. Yiming Ren’s study of lactobacillus protease hydrolysis of soy protein revealed that peptides comprising 10–20 amino acids comprised 72.31–74.08% of the hydrolysis products [48]. In contrast, the oligopeptides released by Apa1 hydrolysis had a lower molecular weight and were hydrolyzed more thoroughly. All the above hydrolysis results show that Apa1 cleaves the protein macromolecules in soybean into low molecular weight polypeptides and free amino acids, which fully reflect the hydrolytic properties of aspartic protease for proteins, facilitate the complete degradation of proteins in the gastrointestinal tract of animals, promotes the digestion and absorption of nutrients such as low molecular weight polypeptides and free amino acids, thereby significantly improving feed utilization and reducing the stimulation of the gastrointestinal tract in feeding young animals [16], and is a promising strategy for improving the digestibility of animal feed.

## Conclusions

In this study, an aspartic protease Apa1 from *A. niger* was expressed for the first time in *P. pastoris*, and a high expression level of 1478.45 U/mL was achieved in a 3 L bioreactor. Apa1 exhibited maximal activity at 50 °C and pH 3.0. It was stable at 30–60 °C and pH 2.0–5.0. Its *K*_m_ and *V*_max_ were 2.44 mg/mL and 83.54 U/mg, respectively. Apa1 completely degraded the anti-nutritional factor components α′ and α, and it significantly degraded the acidic subunit in SPI, which showed that Apa1 was a protease with the highest DH to SPI among the reported aspartic proteases. In summary, the recombinant acidic protease Apa1 had very good properties and was suitable for application in the breeding industry. It could significantly enhance the protein utilization of feed and promote animal health growth. In addition, the large number of small-molecule oligopeptides produced by the degradation of SPI showed that Apa1 had good potential applications in the preparation of soybean peptides.

## Supplementary Information

**Additional file 1: Figure S1.** Alignment of nucleotide sequence between the synthetic gene (upper, *apa1*) and the native gene (lower, *apa*). Mismatched nucleotides were marked with “★”. **Figure S2.** Identification of expression plasmids for *P. pastoris* by enzyme digestion. M: DNA marker; 1-2: product of double enzyme digestion (1122 bp).

## Data Availability

All datasets generated for this study are included in the manuscript.

## Abbreviations

*P. pastoris*: *Pichia pastoris*
SPI: Soybean isolate protein
DH: Hydrolysis degree
*E. coli*: *Escherichia coli*
Zeocin: Bleomycin
YPD: Yeast extract peptone dextrose
YP: Yeast extract peptone
SDS-PAGE: SDS-polyacrylamide gel electrophoresis
PTM1: Trace elements
OPA: O-phenylene formaldehyde method
Mw: Molecular weights
