# Supplementary material for: Purification and characterization of aspartic protease from Aspergillus niger and its efficient hydrolysis applications in soy protein degradation
Source: Microb Cell Fact. 2023 Mar 3;22:42. doi: 10.1186/s12934-023-02047-9 (PMC9983247; doi:10.1186/s12934-023-02047-9)
Supplement: Supplementary file 1 — Additional file 1: Figure S1. Alignment of nucleotide sequence between the synthetic gene (upper, apa1) and the native gene (lower, apa). Mismatched nucleotides were marked with “★”. Figure S2. Identification of expression plasmids for P. pastoris by enzyme digestion. M: DNA marker; 1-2: product of double enzyme digestion (1122 bp). [file 12934_2023_2047_MOESM1_ESM.docx]

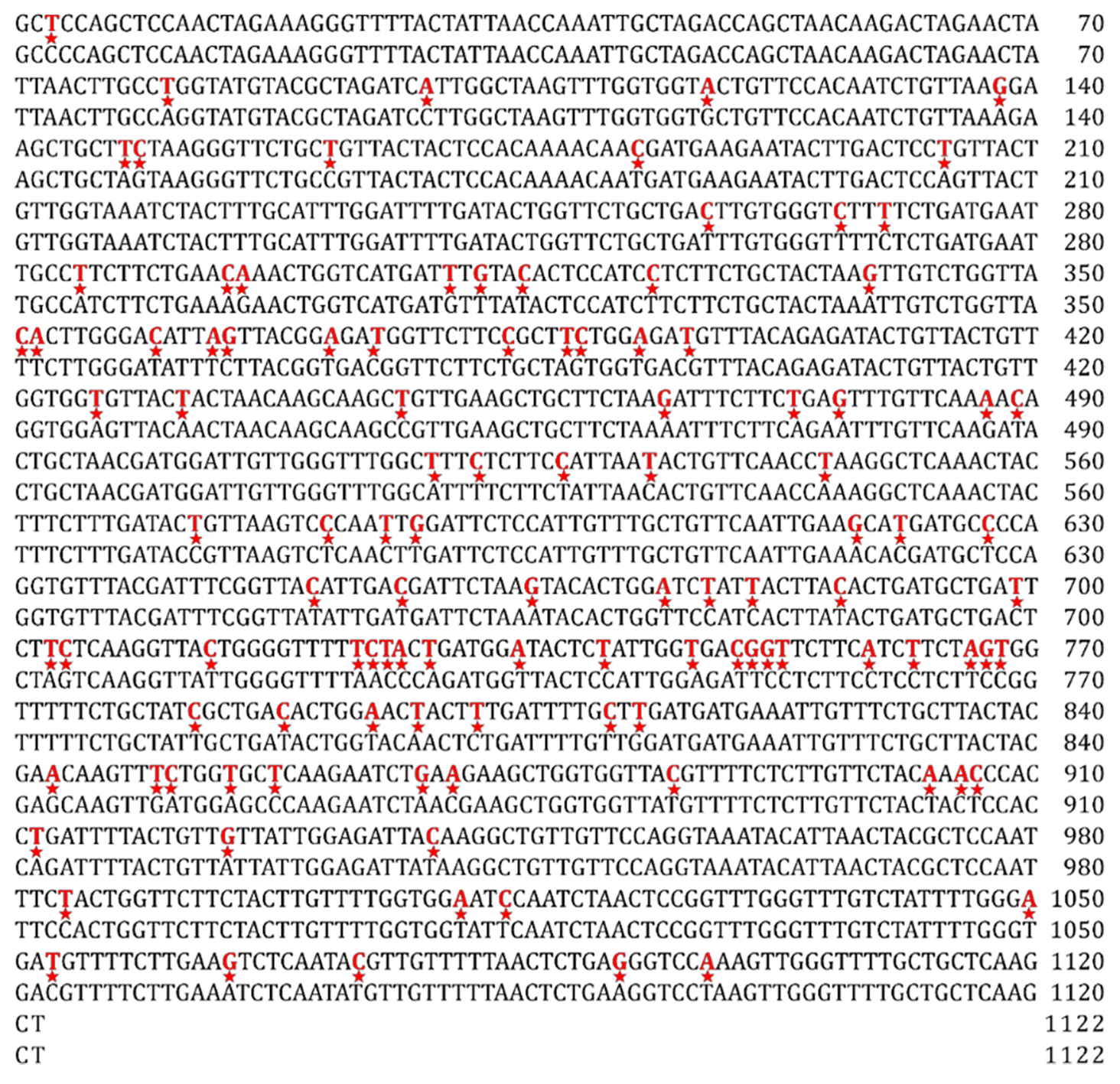


Figure S1. Alignment of nucleotide sequence between the synthetic gene (upper, *apa1*) and the native gene (lower, *apa*). Mismatched nucleotides were marked with “★”.


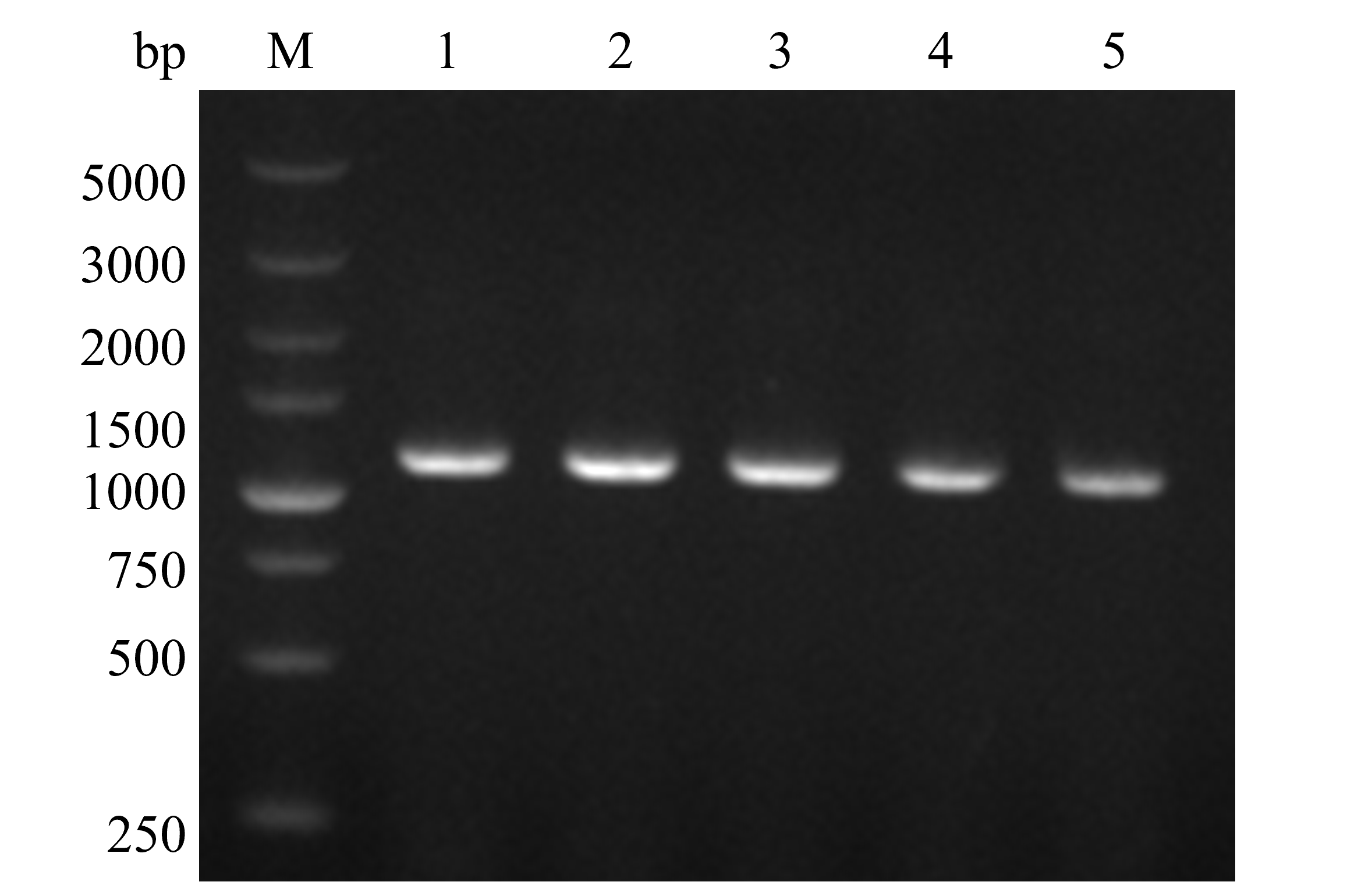


Figure S2. Identification of expression plasmids for *P. pastoris* by enzyme digestion. M: DNA marker; 1-2: product of double enzyme digestion (1122 bp).
